# Effects of Mixtures of Emerging Pollutants and Drugs on Modulation of Biomarkers Related to Toxicity, Oxidative Stress, and Cancer

**DOI:** 10.3390/metabo14100559

**Published:** 2024-10-17

**Authors:** Simona Manuguerra, Fabrizia Carli, Egeria Scoditti, Andrea Santulli, Amalia Gastaldelli, Concetta Maria Messina

**Affiliations:** 1 Laboratory of Marine Biochemistry and Ecotoxicology, Department of Earth and Marine Sciences DiSTeM, University of Palermo, Via G. Barlotta 4, 91100 Trapani, Italy; simona.manuguerra@unipa.it (S.M.); andrea.santulli@unipa.it (A.S.); 2 Institute of Clinical Physiology, National Research Council, 56124 Pisa, Italy; fabriziacarli@cnr.it; 3 Institute of Clinical Physiology, National Research Council, 73100 Lecce, Italy; egeria.scoditti@cnr.it

**Keywords:** cadmium chloride, carbamazepine, polybrominated diphenyl-ether, oxidative stress, HepG2, biomarkers

## Abstract

**Background/Objectives:** Over time, the scientific community has developed a growing interest in the effects of mixtures of different compounds, for which there is currently no established evidence or knowledge, in relation to certain categories of xenobiotics. It is well known that exposure to pollutants causes oxidative stress, resulting in the overproduction of reactive oxygen species (ROS), which can affect signaling pathways that regulate the cell cycle, apoptosis, energy balance, and cellular metabolism. The aim of this study was to investigate the effects of sub-lethal concentrations of mixtures of emerging pollutants and pharmaceuticals on the modulation of biomarkers related to toxicity, oxidative stress, and cancer. **Methods:** In this study, the hepatoma cell line HepG2 was exposed to increasing concentrations of polybrominated diphenyl ether 47 (BDE-47), cadmium chloride (CdCl_2_), and carbamazepine (CBZ), both individually and in mixtures, for 72 h to assess cytotoxicity using the MTT assay. The subsequent step, following the identification of the sub-lethal concentration, was to investigate the effects of exposure at the gene expression level, through the evaluation of molecular markers related to cell cycle and apoptosis (*p53*), oxidative stress (*NRF2*), conjugation and detoxification of xenobiotics (*CYP2C9* and *GST*), DNA damage (*RAD51* and *γH2AFX*), and SUMOylation processes (*SUMO1* and *UBC9*) in order to identify any potential alterations in pathways that are normally activated at the cellular level. **Results:** The results showed that contaminants tend to affect the enzymatic detoxification and antioxidant system, influencing DNA repair defense mechanisms involved in resistance to oxidative stress. The combined effect of the compounds at sub-lethal doses results in a greater activation of these pathways compared to exposure to each compound alone, thereby exacerbating their cytotoxicity. **Conclusions:** The biomarkers analyzed could contribute to the definition of early warning markers useful for environmental monitoring, while simultaneously providing insight into the toxicity and hazard levels of these substances in the environment and associated health risks.

## 1. Introduction

The risk of combined exposure to multiple compounds has become an ongoing challenge for environmental and human health research in recent years [1]. The majority of studies have concentrated on the effects of individual compounds. However, given that humans and wildlife are simultaneously exposed to complex mixtures of contaminants that may interact and produce abnormal effects compared to exposure to single compounds, with cumulative, synergistic, or antagonistic effects, depending on the nature of the toxicant and its mechanism of action [2,3], it is crucial to investigate the effects of contaminant mixtures on human health [4,5].

A growing number of scientific studies have shown that some chemical contaminants, individually or in mixture form, can exert toxicologically relevant effects even at doses much lower than expected [2,3,6,7]. In addition to the dose, the timing of exposure is also a crucial factor in determining the severity of a particular effect [8]. However, the mechanisms and effects of combined chemical contaminants are still poorly understood [9,10].

Several environmental pollutants have been reported to have the ability to disrupt the prooxidant/antioxidant balance within cells, thereby inducing oxidative stress [2,3,6,7]. This is achieved through the formation of reactive oxygen species (ROS), including hydrogen peroxide (H_2_O_2_) and superoxide anion. Oxidative stress is an imbalance between the production of ROS and the cellular ability to detoxify the reactive intermediates or readily repair the resulting damage. The toxic effects are caused by the production of peroxides and free radicals, which damage all components of the cell, including proteins, lipids, DNA, and RNA [11]. In addition to damaging macromolecules, ROS affect molecular and biochemical processes and disrupt the function of DNA repair proteins [12,13], opening biochemical pathways that can have detrimental effects on homeostasis, energy management, metabolisms, and cellular integrity and functions [13].

ROS play a key role in the body’s physiological and adaptive/defensive responses. However, excessive production of free oxygen radicals has the potential to cause tissue damage [3,14]. Therefore, oxidative stress and DNA damage have been proposed as biochemical mechanisms linking exposure to chemical contaminants to adverse health effects such as immunodepression, cancer, and neurological diseases [12].

There is compelling evidence that oxidative stress and ROS play an important role in the etiology and/or progression of a number of human diseases [15,16].

The susceptibility of an individual to specific contaminants may be defined by a variety of factors such as differences in absorption, distribution, metabolism (toxification and detoxification), and excretion of the toxicant, or repair of the damage introduced into DNA, as well as changes in genes controlling protein expression or cell growth and differentiation [16]. Carcinogenesis is a multistep process involving the transformation, survival, proliferation, invasion, angiogenesis, and metastasis of the tumor and may take up to 30 years [17]. The pathways associated with this process have been linked to chronic inflammation, a major mediator of tumor progression [15,16].

Among the various relevant chemical contaminants, flame retardants, heavy metals, and different drugs are the most representative. In this respect, the effects of PBDEs have been evaluated in several experiments both in vivo and in vitro, using different model systems highlighting the crucial role of oxidative stress [3,18,19]. It is clear that these compounds can exert adverse effects at the cellular and molecular levels, ranging from toxicity, immune, reproductive and neurological system impairment, endocrine disruption, and tumors [3,18,19].

The toxicity and subsequent threat to human health from heavy metals (copper, cadmium, mercury, zinc, chromium, etc.) depend on the concentration/bioaccumulation in human tissues [20]. Frequently non-biodegradable heavy metals accumulate in living tissues through the food chain [21]. In addition, in the environment, many drugs are found not only as active molecules but also as metabolites and biotic or abiotic transformation products [22]. Therefore, prolonged exposure may lead to unwanted effects (toxic or disruptive effects) on living organisms [23].

Nowadays, several studies have demonstrated the exposure of humans and wildlife to various chemical contaminants, but these studies have primarily been developed with one type of chemical contaminant [24]. Most combined toxicities are not simply the sum of the effects of single contaminants but rather the synergistic or antagonistic effects of multiple contaminants [5].

A more recent interest of the scientific community is in the effects determined by mixtures of compounds of various kinds, on which there is no established evidence and knowledge, with respect to certain categories of xenobiotics. In this regard, in order to investigate the effects of multiple pollutants, the aim of this study was to evaluate the effect of sub-lethal doses of mixtures of emerging pollutants and drugs (BDE-47, cadmium chloride (CdCl_2_), and carbamazepine (CBZ)) on the human hepatoma cell line (HepG2). The advantage of HepG2 cells is their inherent metabolic capacity of xenobiotic metabolism and their common use in toxicological studies [4,25,26,27].

In addition, the modulation of biomarkers related to toxicity, oxidative stress, and cancer was investigated at the gene expression level in HepG2 cells, through the analysis of genes related to the processes of conjugation and detoxification of substances, DNA damage, and apoptosis, in order to identify possible alterations in the pathways normally activated at the cellular level and to contribute to the definition of early warning markers useful for environmental monitoring and, at the same time, to the identification of the toxicity and hazard levels of these substances in the environment.

## 2. Materials and Methods

### 2.1. Cell Culture Condition and Treatment

The hepatoma cell line (HepG2) (ECACC N° 85011430, Sigma^®^ Sigma-Aldrich, Saint Louis, MO, USA) was routinely cultured in a humidified atmosphere of 5% CO_2_ at 37 °C. Cells were grown in 75 cm^2^ flasks in RPMI 1946 (Sigma-Aldrich, Saint Louis, MO, USA) supplemented in 10% fetal bovine serum (FBS), 2 mM L-glutamine, and 100 μg/mL penicillin–streptomycin. All reagents were from Sigma-Aldrich, Saint Louis, MO, USA. The cells were seeded in 96-well plates at a density of 10^4^ cells/well (Nunc, Darmstadt, Germany) and incubated for 24 h before exposure to chemical contaminants.

In order to verify the cellular cytotoxicity effect, three experiments were conducted: the first trial was carried out to evaluate the dose-dependent toxicity of the individual compounds (BDE-47, CBZ and CdCl_2_) at concentrations in the range of 1 nM–100 µM. The second experiment evaluated the dose-dependent toxicity of a mixture of two compounds (CBZ + BDE-47 and CdCl_2_ + BDE-47) at the same concentrations used for the individual compounds, with the addition of a sub-lethal dose of BDE-47 (1 µM). The last step was the evaluation of the cytotoxic effect of a combination of three compounds (BDE-47-CBZ-CdCl_2_). The mixtures were 1:10:10 µM, 1:50:50 µM, and 1:100:100 µM, for BDE-47, CBZ, and CdCl_2_, respectively.

In all experiments, cells were exposed (six replicates) to different concentrations of BDE-47, ClCd_2,_ and CBZ according to the standardized protocol [2], for 24, 48, and 72 h. Control samples received the same volume of culture medium, and a solvent control of 0.1% (v:v) dimethyl-sulfoxide (DMSO, Sigma-Aldrich, Saint Louis, MO, USA) in media was also included, although the absence of the effects by the vehicle is well known [3].

The PBDE standard was provided by SPECTRA (Rome, Italy); a stock solution of BDE-47 at a concentration of 25 mM was prepared by dissolving the powder compounds in DMSO. Carbamazepine (CBZ) and cadmium chloride (CdCl_2_) were obtained from Sigma-Aldrich, Saint Louis, MO, USA (C4024 and 202908, respectively); stock solutions of CBZ and CdCl_2_ at 25 mM were prepared by dissolving the powder compounds in DMSO and phosphate buffer saline solution (PBS, Sigma-Aldrich, Saint Louis, MO, USA), respectively.

Viability was determined by the 3-(4,5-Dimethythiazol-2-yl)-2,5-triphenyl tetrazolium bromide (MTT) assay (Sigma-Aldrich, Saint Louis, MO, USA) [28]. The optical densities (ODs) at 570 nm with background subtraction at 690 were determined in a microplate reader (Multiskan-Sky Microplate Reader, Thermo-Scientific^TM^, Waltham, MA, USA). Viability was calculated as the percentage absorbance of the sample compared to the absorbance of the control.

### 2.2. Real-Time Reverse Transcription Polymerase Chain Reaction (RT-PCR)

HepG2 cells (500.000 cells/well) were exposed to sub-lethal doses of BDE-47 (1 µM), CBZ + BDE-47 (1:10 μM), CdCl_2_ + BDE-47 (1:10 μM), and a mixture (Mix) of the three compounds (1:10:10 μM BDE-47, CBZ, and CdCl_2_, respectively) for 72 h. Then, the medium was removed, cells were washed with PBS, and 1 mL of PureZOL (Bio-Rad, Hercules, CA, USA) was added to the well.

Total cellular RNA was isolated from the samples in PureZOL using Aurum Total RNA Fatty and Fibrous Tissue Kit (Bio-Rad, Hercules, CA, USA), and the total RNA (1 μg) was reverse-transcribed into complementary DNA (cDNA) using the 5x iScript Reaction Mix Kit (Bio-Rad, Hercules, CA, USA). The amplification was performed in a total volume of 20 μL, containing 0.4 μM of each primer, cDNA diluted 1:10 of the final reaction volume, 1x IQ SYBR Green Supermix (Bio-Rad, Hercules, CA, USA), and nuclease-free water. Amplification was performed in a gradient cycler (C1000 Touch Thermal Cycler, Bio-Rad, Hercules, CA, USA) according to the manufacturer’s protocol.

The relative quantification of genes [*p53*, *NRF2*, *CYP2C9*, *GST*, *SUMO1*, *UBC9*, *RAD51* and *γH2AFX*] was evaluated after normalization with the reference genes. Data processing and statistical analysis were performed using CFX Manager 3.1 Software (Bio-Rad, Hercules, CA, USA). The primers used are shown in Table 1. The relative expression of all genes was calculated by the 2^−ΔΔCT^ (Livak) method [29] using *Homo sapiens β-actin* and *GAPDH* as the endogenous references.

### 2.3. Statistical Analysis

Statistical differences among the groups were assessed by one-way ANOVA analyses, followed by the Bonferroni or Games–Howell test, depending on the homogeneity of the variables. The normality of the variables was confirmed by the Shapiro–Wilk test and the homogeneity of variance by the Levene test. The significance level was 95% in all cases (*p* < 0.05). All the data were analyzed by the computer application SPSS for Windows^®^ (version 20.0, SPSS Inc., Chicago, IL, USA).

## 3. Results

### 3.1. Cytotoxicity as Measured by MTT Assay

**Figure 1:**
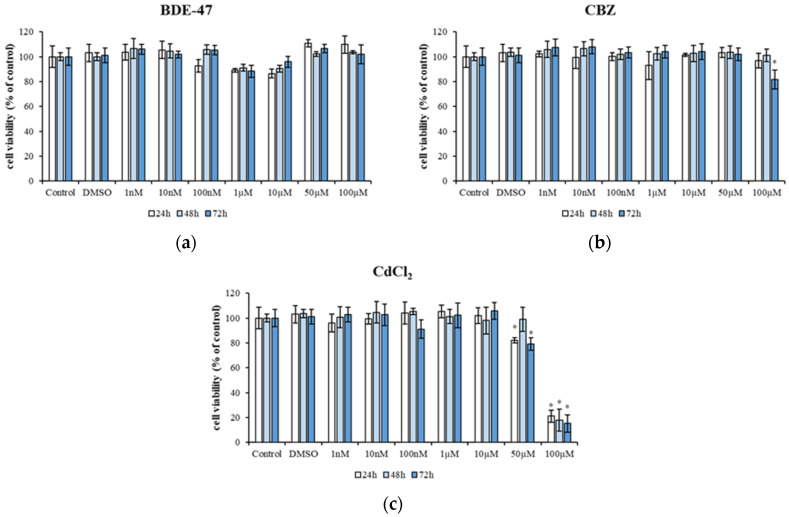
Cytotoxicity of HepG2 cells exposed to different concentrations of BDE 47 (1 nM–100 µM) (**a**), CBZ (1 nM–100 µM) (**b**), and CdCl_2_ (1 nM–100 µM) (**c**) for 24 h, 48 h, and 72 h. Bars represent the mean ± SEM (*n* = 6). Statistical differences (*p* < 0.05) between groups compared to control are indicated by “*”. BDE-47: polybrominated diphenyl ether 47, CdCl_2_: cadmium chloride, CBZ: carbamazepine.

A preliminary cytotoxicity test of each individual compound (BDE-47, CBZ, and CdCl_2_) was performed in the HepG2 cell line using the MTT assay. The cells were exposed to a range of concentrations, from 1 nM to 100 µM, of each chemical compound. No differences were observed in HepG2 cells exposed to single BDE-47 (1 nM to 100 µM) up to 72 h compared to untreated cells (Control) (Figure 1a).

A reduction in cell viability was observed after 72 h of exposure to the highest concentration of CBZ, resulting in a viability of 81.63 ± 7.53% compared to control (*p* < 0.05) (Figure 1b). However, exposure to 50 µM CdCl_2_ resulted in a cell viability of 82.05 ± 2.1% after 24 h, 79.16 ± 4.95 after 72 h (*p* < 0.05). HepG2 cells exposed to 100 µM CdCl_2_ resulted in a cell viability of 21 ± 4.74% after 24 h, 17.95 ± 8.91% after 48 h, and 15 ± 6.88% after 72 h (*p* < 0.05) (Figure 1c).

**Figure 2:**
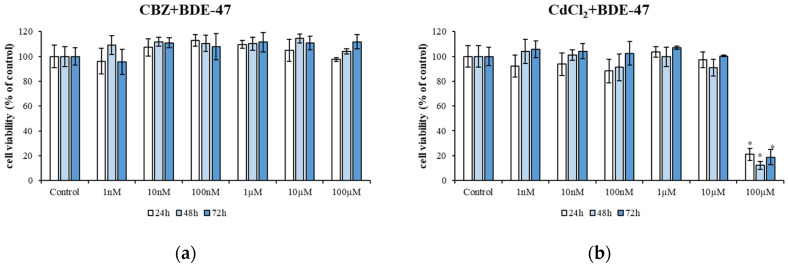
Cytotoxicity of HepG2 cells exposed to different concentrations of a mixture of CBZ (1 nM–100 µM) + BDE-47 1 μM (**a**) and of a mixture of CdCl_2_ (1 nM–100 µM) + BDE-47 1 μM (**b**) for 24 h, 48 h, and 72 h. Bars represent the mean ± SEM (*n* = 6). Statistical differences (*p* < 0.05) between groups compared to control are indicated by “*”. BDE-47: polybrominated diphenyl ether 47, CdCl_2_: cadmium chloride, CBZ: carbamazepine.

The next step was to investigate the effects of a mixture of two compounds on HepG2 cell viability. The cells were exposed to increasing concentrations of CBZ (from 1 nM to 100 µM) (Figure 2a) and CdCl_2_ (from 1 nM to 100 µM) (Figure 2b) mixed with 1 µM BDE-47 for 72 h. No significant reduction in cell viability was observed at different concentrations and times (Figure 2a,b). The highest dose (100 µM) of the CdCl_2_ + BDE-47 mixture demonstrated significant toxicity after 24 h (20.99 ± 4.74% of viability; *p* < 0.05), 48 h (12.14 ± 3.22% of viability; *p* < 0.05), and 72 h (18.80 ± 6.22% of viability; *p* < 0.05) of treatment (Figure 2b) compared to untreated cells.

**Figure 3:**
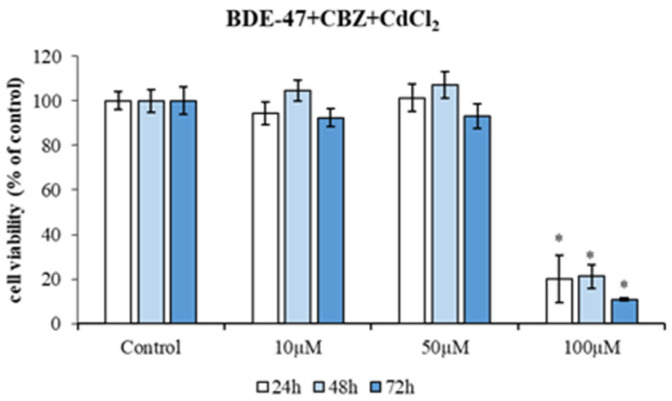
Cytotoxicity of HepG2 cells exposed to different concentrations of a mixture of contaminants (1:10:10 μM; 1:50:50 μM and 1:100:100 μM of BDE-47, CBZ and CdCl_2_, respectively) for 24 h, 48 h, and 72 h. Bars represent the mean ± SEM (*n* = 6). Statistical differences (*p* < 0.05) between groups compared to control are indicated by “*”. BDE-47: polybrominated diphenyl ether 47, CdCl_2_: cadmium chloride, CBZ: carbamazepine.

After the selection of appropriate contaminant doses, HepG2 cells were exposed to increasing concentrations of mixtures of the three compounds: BDE-47 (10 μM) + CBZ (10, 50, and 100 μM) and CdCl_2_ (10, 50, and 100 μM), for a period of 72 h (Figure 3). The cells exposed to the mixture of three compounds exhibited a markedly diminished viability with the highest dose (100 µM) at 24 h (20.13 ± 10.69% of viability; *p* < 0.05), 48 h (21.22 ± 5.34% of viability; *p* < 0.05), and 72 h (10.80 ± 0.60% of viability; *p* < 0.05) of treatment (Figure 3).

### 3.2. Gene Expression Analysis

**Figure 4:**
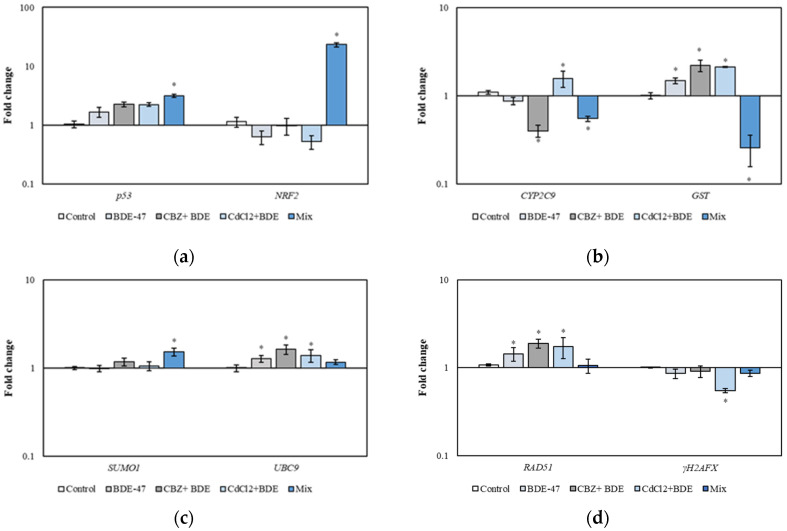
Relative expression of genes related to cell cycle (*p53*) and oxidative stress (*NRF2*) (**a**), detoxification process (*CYP2C9* and *GST*) (**b**), SUMOylation processes (*SUMO1* and *UBC9*) (**c**), and DNA damage repair process (*RAD51* and *γH2AFX*) (**d**) in HepG2 cells exposed to vehicle (Control), 1 μM of BDE-47 (BDE-47), 1 µM di BDE 47 + 10 μM of CBZ (CBZ + BDE), 10 μM of CdCl_2_ + 1 µM di BDE 47 (CdCl_2_ +BDE), and a mixture of contaminants (1 μM of BDE-47+ 10 µM CBZ +10 µM CdCl_2_) (Mix) for 72 h. Statistical differences (*p* < 0.05) between groups compared to control are indicated by “*”. BDE-47: polybrominated diphenyl ether 47, CdCl_2_: cadmium chloride, CBZ: carbamazepine.

The analysis of some relevant genes related to the cell cycle (*p53*), oxidative stress (*NRF2*), detoxification process (*CYP2C9* and *GST*), DNA damage (*RAD51* and *γH2AFX*), and SUMOylation processes (*SUMO1* and *UBC9*) was conducted in HepG2 cells exposed for 72 h to single compound BDE-47 (1 μM), a mixture of two compounds (1 µM of BDE-47+ 10 μM of CBZ and 1 µM of BDE-47+ 10 μM of CdCl_2_), and a mixture of three compounds (1 μM of BDE-47 + 10 μM of CBZ + 10 μM of CdCl_2_) (Figure 4).

The expression of *p53* was significantly increased by 3.14-fold in cells exposed to the mixture of the three compounds (Mix) compared to the control group (*p* < 0.05) (Figure 4a). A significant 23.27-fold upregulation of *NRF2* was observed in cells exposed to the mixture of the three compounds (Mix) compared to the control group (*p* < 0.05) (Figure 4a). The expression of *CYP2C9* was significantly downregulated by 2.5-fold in cells exposed to the mixture CBZ + BDE-47 and by 1.82-fold in cells exposed to the mixture of the three compounds (Mix) compared to the control group (*p* < 0.05). In cells exposed to the mixture CdCl_2_ + BDE, the *CYP2C9* gene exhibited a significant 1.57-fold increase compared to the control (*p* < 0.05) (Figure 4b).

The expression of *GST* was significantly upregulated in cells exposed to BDE-47 (1.49-fold), to the mixture CBZ + BDE-47 (2.21-fold), and to the mixture CdCl_2_ + BDE-47 (2.12-fold) (*p* < 0.05) (Figure 4b). In cells exposed to the mixture of the three contaminants, the expression of *GST* was downregulated by 4-fold (Mix) compared to the control group (*p* < 0.05) (Figure 4b). *SUMO1* gene expression was significantly upregulated by 1.53-fold after Mix treatment compared to the control group (*p* < 0.05) (Figure 4c). *UBC9* expression showed a significant induction in cells exposed to BDE-47 (1.28-fold), CBZ + BDE-47 mixture (1.63-fold), and CdCl_2_ + BDE-47 mixture (1.39-fold) compared to the control group (*p* < 0.05) (Figure 4c). The expression of *RAD51* was upregulated in cells exposed to BDE-47 compound (1.44-fold), CBZ + BDE-47 mixture (1.89-fold), and CdCl_2_ + BDE-47 mixture (1.73-fold) compared to the control group (*p* < 0.05) (Figure 4d). Finally, the expression of *γH2AFX* showed a significant 14.28-fold downregulation in cells exposed to the CdCl_2_ + BDE-47 mixture compared to the control group (*p* < 0.05) (Figure 4d).

## 4. Discussion

In recent decades, environmental contamination by flame retardants, heavy metals, and drugs has become a topic of primary importance for ecosystems and human health [2,11,30,31].

It is known that exposure to contaminants causes oxidative stress, resulting in overproduction of ROS. This can affect signal transduction pathways that regulate a number of cellular processes, including cell cycle, apoptosis, energy balance, and metabolism [2,3,11,18,32]. In support of this consideration, in a previous study on SAF-1 cells, we pretreated the cells with standard antioxidants such as β-carotene and GAE (gallic acid) and then with a mixture of BDE-47, CdCl_2_, and CBZ [2]. The results of viability suggested that antioxidants were able to prevent the effect of exposure to the mixture.

The effects of individual compounds, including 2,2′,4,4′-tetrabromodiphenyl ether (BDE-47), the most abundant flame retardant in the environment, are well documented. Polybrominated diphenyl ethers (PBDEs) constitute a category of lipid-soluble, endocrine-disrupting environmental chemicals. These are synthetic flame retardants that were extensively utilized in numerous commercial products. Flame retardants are chemical compounds that are incorporated into flammable petroleum-based polymeric materials with the objective of reducing the risk of fire and combustion [2,3,11,18,19]. BDE-47 is a highly persistent and easily bioaccumulated carcinogenic flame retardant. It is regarded as one of the most prevalent contaminants in the marine environment, with concentrations ranging from ng/g to μg/g [2,3,11,18,19].

The discharge of human industrial activities into the global environment, particularly into soil, water, and air, has a significant impact on the distribution of metals worldwide. Cd is a metal that accumulates in plants, animals, and soil, and represents a significant hazard to human health [11,20]. The presence of pharmaceuticals in urban wastewater, including CBZ has recently emerged as a new environmental concern [33,34,35].

The mechanism underlying the toxic effects of chemical contaminants remains unclear, particularly at low doses. However, the literature indicates that exposure to realistic doses of contaminants such as PBDE could result in a bimodal response in cellular systems. This suggests that low concentrations of compounds may promote cell proliferation, while high concentrations of compounds may inhibit cell growth [2,3,36].

In this study, we carried out a preliminary exposure to increasing concentrations of BDE-47, CBZ, and CdCl_2_ in HepG2 cells to test the effects on cell viability for a short period of time (24, 48, and 72 h). Then, after the sub-lethal dose was chosen, the effects of the mixtures of the compounds were analyzed in order to investigate how these contaminants affect gene expression involved in different cellular pathways [2,3,18].

The preliminary test results showed that exposure to single rising doses of BDE-47, CBZ, and CdCl_2_ did not reduce cell viability. However, the higher concentrations of CBZ (100 µM) and CdCl_2_ (50–100 µM) induced inhibition of cell proliferation in a concentration-dependent manner. Our findings correlate with those of Wang et al. [34], showing that elevated BDE-47 concentrations elicited a dose-dependent inhibition of cell proliferation in HepG2 cells, manifesting as hormesis. This phenomenon is characterized by a biphasic dose-response, wherein low-dose exposure elicits beneficial effects, while high-dose exposure results in toxic effects, as observed in single chemical or mixture exposure scenarios [36,37]. Similar results were obtained by Manuguerra et al. [3] in human fibroblasts (HS-68) exposed to increasing concentrations of BDE-47; in fact, only at the highest doses the cells showed a significant decrease in viability. An in vitro study on the human monocytic leukemia cell line (THP-1) demonstrated that BDE-47 concentrations (ranging from 3 to 25 µM) did not significantly reduce viability after 24 and 48 h [38]. In contrast, studies reported a significant decrease in cell viability in neuronal stem cells treated with PBDE at lower concentrations [39,40]. However, the discrepancies observed may be attributed to the type of cell system employed [39,40].

As previously described, CBZ did not significantly reduce the cell viability of HepG2 cells after treatment with increasing concentrations (Figure 1), indicating that CBZ at the concentrations tested is unable to inhibit cell growth. In the murine liver, CBZ was demonstrated to promote hepatocyte proliferation, thereby reducing cell death through the induction of survivor signals [41].

In contrast with the present results, Sohaib and Ezhilarasan [42] reported that treatment with CBZ caused significant and concentration-dependent cytotoxicity in human colon cancer cells (HT-29). Similar results were also obtained by Akbarzadeh et al. [43], who observed that colon cancer cell (SW480) treatment with CBZ inhibited cell growth in a dose-dependent manner.

The administration of CdCl_2_ resulted in a notable decline in cell viability, as illustrated in Figure 1. This decline was dose- and time-dependent. This finding is consistent with the observations of other authors who demonstrated in vitro cytotoxic effects of CdCl_2_ [44,45].

Oxidative stress is a pivotal factor in CdCl_2_-induced toxicity, leading to cellular damage through the overproduction of ROS. This was evidenced in a study conducted on cancer cells, wherein CdCl_2_ promoted oxidative stress, resulting in DNA damage and programmed cell death [46,47].

With regard to the combined effect of the two compounds, only the highest dose for the CdCl_2_ + BDE-47 mixture showed a significant decrease in viability (Figure 2), highlighting the toxic effect of cadmium. This result was further confirmed by the exposure of cells to the mixture of the three compounds (Mix), in which the highest dose, until 72 h, induced a significant reduction in viability (Figure 3), suggesting the preponderant role of CdCl_2_ in inducing a cytotoxic effect.

Nevertheless, at lower concentrations of the mixture, no significant decrease in viability was observed (Figure 3). As previously outlined, this phenomenon may be related to the phenomenon of hormesis [48], whereby a toxic substance at lower concentrations shows a high affinity for specific target sites but is unable to induce cell death. The binding of a compound to a putative target within the cell can activate certain cellular defense mechanisms that modulate and reduce the toxicity of other compounds [48]. Furthermore, the reduction in overall toxicity may be attributed to the inability of more toxic compounds to bind to the same hypothetical target site [49].

In human hepatoma cancer cells (SMMC-77721), the combination of cadmium with BDE-209 caused a significant reduction in cell viability compared to the individual compounds. Therefore, the treatment with cadmium, a typical toxic heavy metal, in combination with BDE-209, an organic compound that persists in the environment, exacerbates liver injury due to the augmented production of ROS by the combined action of the two contaminants [50]. The data indicate that the observed cytotoxicity may be attributed to a significant increase in oxidative stress, as previously demonstrated in our studies conducted in human (HS-68) and fish (SAF-1) fibroblast cell lines [3,18]. It is well established that a prolonged state of oxidative stress and the depletion of antioxidant capacity can affect cellular homeostasis in a variety of ways, inducing DNA oxidation or damage, thereby facilitating the emergence of mutations and the activation of proliferative processes involved in carcinogenesis [2,3,51]. We did not measure ROS production; therefore the hypothesis of ROS overproduction by the tested contaminants in our experimental condition should be further investigated.

The expression of selected relevant markers related to the cell cycle, oxidative stress, detoxification process, DNA damage, and SUMOylation processes was evaluated in HepG2 cells.

p53 is a central regulator of the cellular response to various forms of stress, genotoxic insults, and DNA damage. It induces cell cycle arrest by inducing damage response genes and causing apoptosis [52,53].

Exposure to a mixture of contaminants severely affects markers related to cell cycle regulation and oxidative stress. Indeed, *p53* expression was upregulated in cells exposed to the mixture of compounds (Mix), indicating that the contaminants may potentially disrupt cellular homeostasis, likely due to oxidative stress induced by the combined effect of the toxicant mixture [2,3,19].

The elevated expression of p53 observed by Song and Koh [54] in human lens epithelial cells (CRL 11241) exposed to CdCl_2_ provides evidence that cadmium influences cytotoxicity and cell death through a p53-dependent pathway. In the metastatic prostate adenocarcinoma cell line (LNCaP) and the primary prostate cancer cell line (22Rv1), treatment with cadmium induced a significant increase in p53 levels, highlighting the capacity of cadmium to induce p53-dependent apoptosis in tumor cells and inhibit cell proliferation [55].

With regard to the effects of contaminants on the oxidative status of HepG2 cells, our results showed a significant increase of *NRF2* mRNA levels in cells treated with a combination of three compounds (Mix), indicating that exposure to this mixture at a sub-lethal dose reveals the detrimental effects of the individual compounds, which stimulates the induction of a defense mechanism against cellular stress. This phenomenon has been shown to intensify the toxicity of the compounds and to stimulate cellular mechanisms that may potentially lead to cell transformation [56]. When activated, NRF2 recruits transcriptional factors that stimulate the expression of target genes involved in antioxidant defense, DNA repair, phase I and II metabolism, and the prevention of apoptosis. Collectively, these processes contribute to the maintenance of a healthy and proliferating cell [57]. These results agree with our previous research on the SAF-1 cell line exposed to the same mixture, which exerted a pronounced effect on cell viability by promoting the expression of genes associated with cell cycle regulation, stress response, cell survival, and oxidative stress [2]. Further evidence that contaminants can induce oxidative stress has been provided by studies on human fibroblasts (HS-68). These studies have demonstrated that exposure to a mixture of flame retardants (BDE-209, BDE-47, and BDE-99) induces a stress response, accompanied by a significant increase in NRF2 expression levels [3].

The expression of phase I (CYP2C9) and II (GST) enzymes was found to be significantly affected by exposure to different chemical contaminants. The cytochrome P450 (CYP450) superfamily is represented by phase I enzymes, particularly monooxygenases, which participate in the metabolism of endogenous substrates and play a pivotal role in the detoxification and metabolic activation of xenobiotics [58]. CYP2C9 plays a significant role in the total microsomal P450 content of the human liver, with the capacity to metabolize over 16% of the drugs and xenobiotics present in the human liver [59].

The expression of *CYP2C9* was significantly downregulated in HepG2 cells exposed to CBZ + BDE-47 and Mix. This lower expression of *CYP2C9* mRNA may be associated with enhanced cellular survival and the development of cancer [60]. Furthermore, it has been demonstrated that flame retardants such as TBBPA (tetrabromobisphenol A) induce a downregulation of CYP2C9, thereby impairing phase I detoxification mechanisms [61].

As previously outlined, oxidative stress results in the activation of NRF2, which is then translocated from the cytoplasm to the nucleus. There, it plays an active role in regulating the induction of phase II enzymes. In the liver, binding of the nuclear factor NRF2 to the antioxidant response element (ARE) is essential for the expression and induction of the *GST* gene [62].

Our results showed that the expression of *GST* is upregulated in cells exposed to CdCl_2_ + BDE-47, with respect to the control group, suggesting that exposure to these kinds of compounds entails oxidative stress. Interestingly, the mixture of the three contaminants (Mix) caused downregulation of the *GST* gene. It is possible that GST may indicate a failure in the process of detoxification and the occurrence of oxidative stress in cells [63]. These observations indicate that chemical contaminants, acting in combination, are capable of inducing an increased oxidative stress condition in the cell, which is likely to impair phase I and II detoxification mechanisms.

In HepG2 cells, *SUMO1* was found to be upregulated in cells exposed to the mixture of the three compounds (Mix), while *UBC9* expression was upregulated after exposure to BDE-47 alone and to CBZ + BDE-47 and CdCl_2_ + BDE-47. The upregulation of *SUMO1* may result in the depletion of cellular free SUMO levels, thereby forcing the cell to express more SUMO, particularly under highly stressed conditions [64]. Modulation in SUMO1 and UBC9 expression was associated with diseases such as neurodegeneration and cancer [65].

SUMOylation is the covalent attachment of the small ubiquitin-related modifier (SUMO) to a vast variety of proteins in order to modulate their function, including replication, DNA damage response, transcription, RNA maturation, and cell cycle. The process of SUMOylation is subject to modulation under conditions of oxidative, osmotic, and hypoxic stress [64,66]. The binding of SUMO to proteins is regulated by an enzymatic cascade involving the heterodimer E1 activating enzyme (SAE1/UBA2), E2 conjugating enzyme (UBC9), and an E3 ligating enzyme [67].

The correlation between oxidative stress and SUMOylation, as demonstrated by Bossis and Melchior [68] through the activation of the enzyme SUMO E2 UBC9 with hydrogen peroxide, provides further confirmation of its role as a sensor of ROS for an early response to oxidative stress.

Rad51 plays a pivotal role in repairing ROS-induced DNA damage, promoting the process of homologous recombination, thus enhancing cellular resistance to oxidative stress and maintaining genomic stability [69,70]. Our results revealed that HepG2 cells exposed to CBZ + BDE-47 and CdCl_2_ + BDE-47 showed an upregulation of genes involved in DNA repair. This suggests that the toxicants generated by these treatments resulted in the formation of DNA double-strand breaks, leading to an increase in *RAD51* gene expression to facilitate DNA damage repair [71,72].

Regarding the *γH2AFX* (a well-known DNA damage indicator) expression, our results contrast with other authors that reported an increased expression of *γH2AFX* in Human Bronchial Epithelial (BEAS-2B) cells after exposure to CdCl_2_ [73] and in the SQ20B cell line derived from human epithelium tumors of the larynx exposed to CdCl_2_ [74]. These observations may indicate that a toxic condition, probably due to oxidative stress as suggested by the upregulation of NRF2 and the impairment of detoxification mechanisms, is responsible for the DNA damage attested by the changes in these markers. However, it appears that CdCl_2_ may selectively have a greater influence on certain DNA repair pathways.

The present study has limitations. Further studies are needed to investigate the effect of mixtures of compounds on the DNA repair system. The human hepatoma cell line HepG2 is a reproducible and easy-to-handle model for studies on xenobiotics metabolism and toxicology [75]. Moreover, hepatoma cells overcome the limitations of using primary hepatocytes, mostly in screening studies, including the limited availability of liver samples, inter-individual variability, limited survival, loss of metabolic activity after long culture, and costs [76,77]. However, the HepG2 cell line is less suitable compared with primary hepatocytes, which more closely resemble the in vivo situation. HepG2 cells express lower levels of phase I metabolism enzymes in contrast with primary hepatocytes [78,79,80], thus decreasing the metabolic capacity of HepG2 cells and rendering them less sensitive to xenobiotics. The effects of contaminants should therefore be validated in human primary cells before drawing conclusions from results obtained in hepatoma cells.

Moreover, further studies should be performed to assess the effects of contaminant mixtures on other tissues and organs in addition to the liver.

## 5. Conclusions

The results highlighted the usefulness of the experimental system and markers used in monitoring the effects of chemicals alone or in combination.

The collective findings of this study confirm that exposure to mixtures caused higher cytotoxicity and alterations in mRNA gene expression in HepG2 cells. During exposure, contaminants, not only alone but also in combination, tend to affect the enzymatic detoxification system, affecting the defense mechanisms of DNA repair involved in resistance to oxidative stress. The observed alterations at the molecular level could contribute to the definition of early warning markers useful for environmental monitoring and, at the same time, help identify the toxicity and hazard levels of these stressors in the environment. Further studies are required to elucidate the relationship between co-exposure to different contaminants and cancer development, as well as the molecular mechanisms linking exposure with oxidative stress.

## Figures and Tables

**Table 1:** Primer sequences used in RT-PCR.

Gene	Access Number	F/R Primer Sequence (5′–3′)
*p53*		AAGAAACCACTGGATGGAGAA CAGCTCGGAACATCTCGAA
*NRF2*		ATAGCTGAGCCCAG TATC CATGCACGTGAGTGCTCT
*CYP2C9*	KF248055.1	AGGCACACACCGAATTAGCA TCTCCCAGAGCTCTGTCTCC
*GST*	M99422.1	GGACGCCTTCCCAAATCTGA CAGTTTGGTGGATGCCTCCT
*SUMO1*		TGTGGGGAAGGGAGAAGGAT AAGGTTTTGCCTCCTGGTCA
*UBC9*		CGAACCACCATTATTTCACC GGATCTGTTTGATTGTGATGG
*RAD51*		GCCCTTTACAGAACAGACTACT AAACATCGCTGCTCCATCC
*γH2AFX*	NM_002105.2	GGTGCTTAGCCCAGGACTTT TGGAGGGAGAGCTGATGTGA
*GAPDH*		ACCCACTCCTCCACCTTTGAC GTCCACCACCCTGTTGCTGTA
*β actin*		AGGCTGTGCTGTCCCTGTAT ACCCAAGAAGGAAGGCTGGA

## Data Availability

Data contained within the article.
